# Cross-cultural adaptation and psychometric validation of the first Arabic KOOS-12: a reliable tool for assessing knee outcomes in Arabic-speaking populations

**DOI:** 10.1186/s43019-026-00316-6

**Published:** 2026-04-10

**Authors:** Marc Boutros, Guy Awad, Caren Hassan, Shaza Hammad, Julia Assi, Gaby Haykal, Toni Mansour

**Affiliations:** 1 Faculty of Medicine, Saint Joseph University, Beirut, Lebanon; 2 Division of Orthopaedic Surgery, Hôtel-Dieu de France, Beirut, Lebanon

**Keywords:** KOOS-12, Arabic translation, Cross-cultural adaptation, Knee osteoarthritis, Patient-reported outcome measures, Reliability, Validity, Psychometrics, WOMAC

## Abstract

**Background:**

The Knee injury and Osteoarthritis Outcome Score-12 (KOOS-12) is a widely used patient-reported outcome measure assessing pain, function, and quality of life (QOL) in individuals with knee osteoarthritis or injury. However, the absence of validated short-form knee instruments in Arabic necessitates cross-cultural adaptation.

**Methods:**

A prospective cross-sectional validation of the Arabic KOOS-12 (KOOS-12 AR) was conducted following established guidelines for translation and adaptation (independent forward translations, reconciliation, blinded back-translation, expert review, pilot testing, and proofreading). Adults > 40 years with knee osteoarthritis were consecutively recruited from two outpatient clinics and completed the KOOS-12 AR and the validated Arabic WOMAC (WOMAC-AR). A total of 201 patients were enrolled, and a clinically stable subset of 91 participants (the retest subgroup) completed a retest after 5–10 days. Psychometric evaluation included interpretability, internal consistency (Cronbach’s *α*), test–retest reliability using intraclass correlation coefficients (ICC(2,1)), and measurement error indices such as standard error of measurement (SEM), minimal detectable change (MDC), and content, structural, and convergent validity.

**Results:**

Internal consistency was excellent across domains (Cronbach’s *α* = 0.93). Test–retest reliability was likewise excellent (ICC(2,1) = 0.990 for Pain, 0.992 for Function, 0.989 for QOL, and 0.987 for the Total score). SEM values were small (2.1–2.3 points), yielding MDC_individual ≈6 points across subscales. Bland–Altman analyses showed minimal bias (−0.36 to −0.02) and narrow limits of agreement (approximately ± 6–7 points). Structural validity was supported by principal component analysis (PCA): each subscale demonstrated a clear one-factor solution (Pain eigenvalue 2.50, 62.5% variance; Function 2.72, 67.9%; QOL 2.48, 62.1%). When all 12 items were analyzed together, the dominant first component explained 50.6% of total variance (Kaiser–Meyer–Olkin (KMO) = 0.91). Convergent validity with WOMAC-AR was strong, with Spearman’s *ρ* = 0.879 for total scores (*p* < 0.0001).

**Conclusion:**

The KOOS-12 AR demonstrated excellent reliability, validity, structural coherence, and measurement reproducibility. It represents a robust, culturally appropriate, and feasible instrument for evaluating knee pain, function, and QOL among Arabic-speaking patients.

**Supplementary Information:**

The online version contains supplementary material available at 10.1186/s43019-026-00316-6.

## Introduction

In today’s medical practice, assessing quality of life (QOL) has become critical and is regarded as a pivotal outcome, especially in orthopedics. Considered equally significant to surgical and postoperative measures, it has become a fundamental parameter for evaluating the effectiveness of an intervention and is increasingly reported in many studies [1–5]. QOL metrics help analyze patients’ perspectives and document their preferences. They therefore represent crucial indicators of patient satisfaction, which may be implicated in long-term outcomes [6]. Moreover, implementable findings can be achieved during recovery through the monitoring of patient-reported outcome measures (PROMs). The literature highlights considerably improved outcomes among individuals who frequently completed PROMs while receiving automated alerts compared with those receiving standard care [7].

It is well established that patients’ personal viewpoints give QOL metrics a highly subjective aspect. Objectifying these metrics becomes pivotal to ensure accurate appraisal and synthesis of evidence, while enabling consistency in measurement and reliability in comparisons across studies. Standardized questionnaires can systematically assess variables such as pain and function by translating them into quantifiable data. They facilitate objective evaluation of patient improvement by clinicians. The Knee injury and Osteoarthritis Outcome Score-12 (KOOS-12) is a shortened version of the original 42-item KOOS. It comprises 12 items organized into three domains: Pain, Function/Daily Living, and knee-related QOL, with four items per domain [8, 9]. It provides a summary knee impact score and three domain-specific scores with markedly reduced respondent burden. The KOOS-12 has also demonstrated excellent responsiveness and good psychometric properties for capturing joint replacement outcomes [8, 10], and it has additionally demonstrated structural validity across its three domains in joint replacement populations [11]. All items of the KOOS-12 refer specifically to the knee; patients are therefore asked to focus on a single joint when evaluating their pain or functional limitations, rather than other orthopedic or nonorthopedic comorbid conditions [9].

To efficiently apply this tool in different cultural and linguistic contexts, correct translation is required to ensure both validity and accuracy. A persistent lack of instruments in non-English-speaking countries represents a central challenge, mandating the need for cross-cultural adaptation. Accurate assessment and rigorous comparison of functional status across diverse populations can be achieved through the adoption of translation, which emerges as a superior approach compared with developing a new questionnaire [12].

This study is the first to translate, culturally adapt, and validate the KOOS-12 into Arabic for patients with knee osteoarthritis. Beyond its regional relevance, this work addresses a broader methodological need in contemporary orthopedic research, in which linguistic equivalence of PROMs is essential to ensure valid cross-cultural comparisons. Arabic is spoken by more than 420 million individuals worldwide, including substantial diaspora populations in North America and Europe, with increasing representation in multinational clinical trials and global registries [13–15]. The absence of a validated Arabic version of the KOOS-12 limits equitable inclusion of Arabic-speaking patients and introduces linguistic bias in outcome assessment, thereby restricting comparability across studies and clinical settings. Accordingly, the objective of this study was to translate, culturally adapt, and psychometrically validate an Arabic version of the KOOS-12 (KOOS-12 AR) for use in Arabic-speaking patients with knee osteoarthritis. We hypothesized that the KOOS-12 AR would demonstrate excellent internal consistency, test–retest reliability, and structural validity, and would show strong convergent validity with an established Arabic knee-specific outcome measure.

## Materials and methods

### Study design and eligibility criteria

A prospective cross-sectional validation study was conducted to evaluate the KOOS-12 AR. The comparator instrument was the Arabic Western Ontario and McMaster Universities Osteoarthritis Index (WOMAC-AR), previously validated [16]. KOOS-12 includes three domains: pain (four items), function/daily living (four items), and knee-related QOL (four items). Each domain was scored according to the KOOS-12 manual and linearly transformed to a 0–100 metric, with higher scores indicating better status. An overall KOOS-12 composite (0–100), defined as the mean of the three subscales, was used secondarily for convergent analyses. WOMAC-AR was scored per its manual, after which pain (/20), stiffness (/8), difficulties (/68), and total (/100) scores were reversed using the formula (maximum possible score—observed score) and linearly rescaled to a 0–100 metric so that higher scores also indicated better status, thereby enabling direct comparability with KOOS-12.

Consecutive patients were recruited over 2 years from the waiting rooms of two outpatient clinics during routine visits, until the target sample size greater than 200 participants was reached.

Participants were eligible for inclusion if they were > 40 years old; had a clinical and/or radiographic diagnosis of knee osteoarthritis (unilateral or bilateral); reported knee pain and/or functional limitation primarily attributable to knee osteoarthritis; were native or fluent Arabic speakers; and were able to provide written informed consent and complete patient-reported outcome questionnaires independently or with minimal assistance. Patients were excluded if they had other primary joint pathology in the index knee (e.g., inflammatory arthritis, crystal arthropathy, septic arthritis), a history of major trauma or surgery to the index knee that could substantially alter symptoms independently of osteoarthritis (including recent periarticular fracture, ligament reconstruction, meniscal surgery, osteotomy, or any previous knee arthroplasty), severe concomitant musculoskeletal or neurological disorders affecting lower limb function (such as advanced hip osteoarthritis, stroke with residual deficit, severe neuropathy, or Parkinson’s disease), cognitive impairment or severe psychiatric illness precluding reliable questionnaire completion, inability to adequately communicate in Arabic, current acute knee injury or acute exacerbation requiring emergency care in the previous 3 months, or participation in another interventional study targeting knee symptoms that was likely to modify pain or function.

### Permissions and authorizations

Permission to translate and adapt the KOOS-12 AR was obtained from MAPI Research Trust (hereafter referred to as MAPI), the international organization responsible for the distribution and licensing of many validated patient-reported outcome measures. MAPI manages the official permissions process for KOOS-12, which allows authorized users to obtain translation rights directly through their system [17]. Similarly, permission to use the validated WOMAC-AR as a comparator was obtained from its authors [16].

### Translation

Cross-cultural adaptation followed established guidelines [18]. Three independent forward translations from English to Arabic were produced by one sworn translator and two practicing knee surgeons, then reconciled into a single forward version. A back-translation into English was performed by a native English translator who was blinded to the original instrument. An expert panel consisting of a statistician or researcher, two knee clinicians, and sworn translators reviewed semantic, idiomatic, experiential, and conceptual equivalence between the back-translation and the source version and resolved all discrepancies. A pilot study with 10 clinic patients assessed comprehensibility, and any misunderstood wording was revised. Final proofreading by all authors produced the Arabic version (KOOS-12 AR).

Details of the cross-cultural adaptation process and item-level comparisons between the original English version and the Arabic version are provided in Supplementary Table 1.

### Study population and characteristics

A total of 201 patients were enrolled. Of these, 91 clinically stable participants constituted the retest subgroup, defined as no important change in knee symptoms and no change in treatment (including no surgery) between assessments, and completed the KOOS-12 AR again 5–10 days later.

Age was not normally distributed (Shapiro–Wilk *p* < 0.0001); median age was 59 years (IQR 44–68). The female-to-male ratio was 136:65, and dominant limb distribution was right:left = 146:55.

### Procedures and data capture

All patients completed both questionnaires, the KOOS-12 AR and the WOMAC-AR, using a standardized Google Form^TM^ after providing informed consent. An investigator was present to offer assistance only if clarification was required.

The retest subgroup was invited to complete the KOOS-12 AR again after 5–10 days. This interval was selected to reduce recall of prior responses while minimizing true clinical change. Contact information was recorded solely for the purpose of this follow-up.

All responses were exported to a single Excel spreadsheet for statistical analysis. Each participant was assigned a unique study code to ensure anonymity, and the analysis dataset contained no directly identifying information.

### Ethical approval and consent

Ethical approval was obtained from the Institutional Review Board of Hôtel-Dieu de France, Beirut (CEHDF-2465). The study adhered to the Declaration of Helsinki, and informed consent was obtained from all participants.

### Statistical analysis

All analyses were conducted in XLSTAT^TM^ (Addinsoft, Paris, France). Two-sided tests were used with *α* = 0.05. Effect sizes and 95% confidence intervals (CI) are reported where applicable. Analyses were performed on complete cases; for factor analysis, listwise deletion was applied.

- Descriptive statistics and interpretability

For KOOS-12 AR subscales (Pain, Function, QOL) and total, and for WOMAC-AR subscales (pain, stiffness, difficulties) and total, score distributions were summarized using minimum, maximum, median, and interquartile range (IQR). Normality was assessed with the Shapiro–Wilk test. Interpretability was examined via floor and ceiling effects, defined as the proportion of respondents at the theoretical minimum or maximum; values < 15% were considered acceptable.

b) Internal consistency

Internal consistency of the KOOS-12 AR Pain, Function, and QOL subscales, as well as the overall KOOS-12 AR composite, was evaluated using Cronbach’s *α* with 95% confidence intervals (CI) corrected item–total correlations (CITC), and “*α* if item deleted.” CITC > 0.30 were considered acceptable, with higher values indicating stronger item–scale coherence. Values of Cronbach’s *α* > 0.70 were regarded as acceptable, > 0.80 as good, and > 0.90 as excellent.

c) Structural validity

Structural validity of the KOOS-12 AR was explored using principal component analysis (PCA) on the 12 items. Sampling adequacy was assessed with the Kaiser–Meyer–Olkin (KMO) statistic and Bartlett’s test of sphericity. Components were extracted using PCA and interpreted based on the scree plot, eigenvalues > 1, and interpretability. Factor loadings > 0.40 were deemed salient.

d) Convergent validity

Convergent validity was examined using Spearman’s rank correlation (*ρ*) between KOOS-12 AR scores and WOMAC-AR scores. Primary analyses assessed correlations between KOOS-12 AR subscales and the corresponding WOMAC-AR subscales (e.g., KOOS-12 Pain vs WOMAC-AR Pain), as well as between the KOOS-12 AR composite and WOMAC-AR Total. Because both instruments yield bounded, ordinal-derived scores with non-normal, tie-prone distributions and an expected monotonic (not strictly linear) relationship, Spearman’s *ρ* was used. Correlations were reported with 95% CI.

e) Short-interval change (5–10 days)

For participants reassessed within 5–10 days, change scores (Δ = Time2−Time1) were summarized as mean Δ ± SD with 95% CI for KOOS-12 AR subscales and Total.

f) Test–retest reliability and measurement error

In the retest subgroup, test–retest reliability of KOOS-12 AR Total (and, secondarily, subscales) was estimated using ICC(2,1) (two-way random effects, absolute agreement, single measures) with 95% CI. Systematic change between Time1 and Time2 was examined using paired tests appropriate to the distribution. Measurement error indices were derived as follows:

- Standard error of measurement (SEM) = SD × √(1 − ICC), using the Time1 SD of the retest subgroup;
- Minimal detectable change for an individual (MDC_individual) = 1.96 × √2 × SEM;
- Minimal detectable change for a group (MDC_group) = MDC_individual/√_n_.

These indices provide thresholds for interpreting observed KOOS-12 AR score changes beyond measurement error at the individual and group levels.

## Results

### Translation and cross-cultural adaptation of the KOOS-12 AR

The finalized version of the KOOS-12 AR, obtained after cross-cultural adaptation, is presented in Supplementary Fig. 1.

### Interpretability

**Fig. 1:**
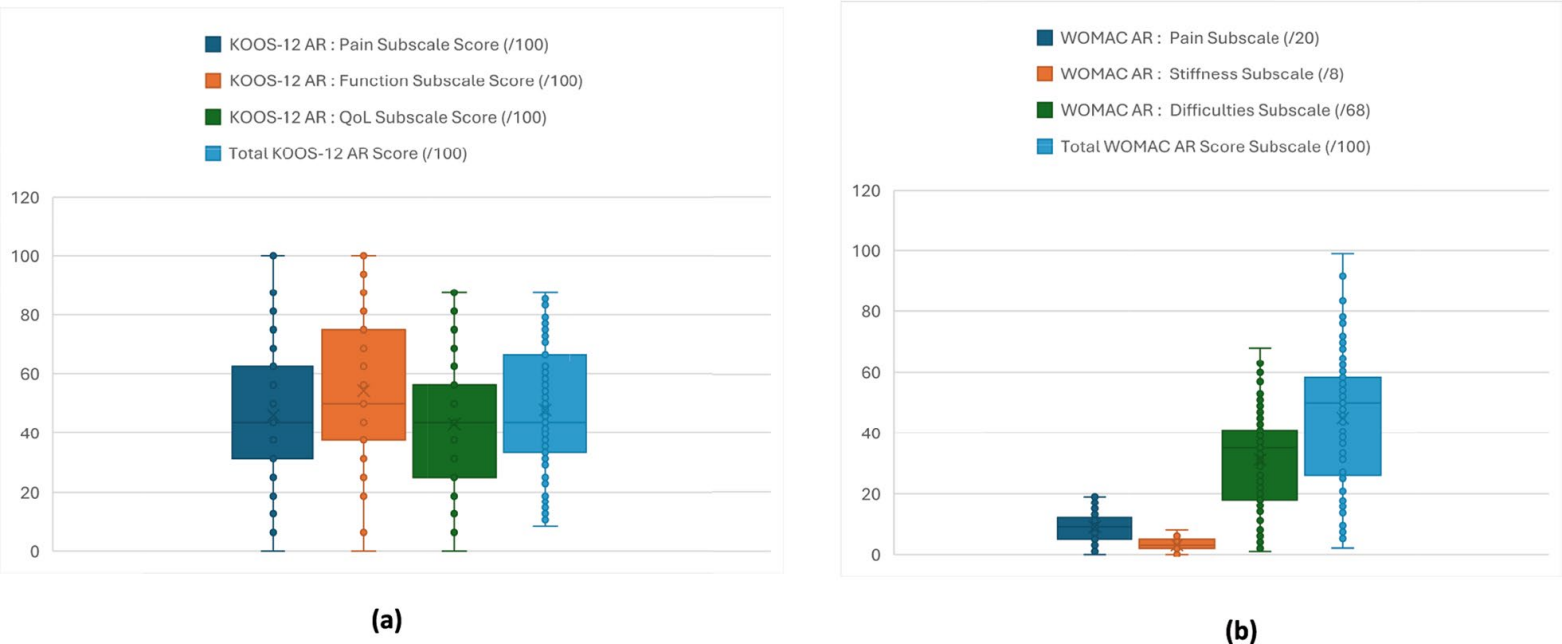
Score distributions for the Arabic WOMAC (WOMAC-AR) and Arabic KOOS-12 (KOOS-12 AR) (*n* = 201): **a** WOMAC-AR—Pain (/20), Stiffness (/8), Difficulties (/68), and Total (/100); **b** KOOS-12 AR—Pain, Function, QOL, and Total (all/100). Box-and-whisker plots show the median (line), IQR (box), and range (whiskers); “ × ” marks the mean

Observed scores spanned the full theoretical range (Table 1). Floor and ceiling effects were ≤ 15% for all KOOS-12 AR and WOMAC scores except the WOMAC Stiffness subscale, which showed a floor of 23.88% with no ceiling effect (likely due to its few items and restricted variability in stiffness) (Fig. 1).

**Table 1:** Descriptive statistics and distributional properties of KOOS-12 AR and WOMAC-AR scores (*n* = 201)

	KOOS-12 AR: pain subscale score (/100)	KOOS-12 AR: function subscale score (/100)	KOOS-12 AR: QoL subscale score (/100)	Total KOOS-12 AR score (/100)	WOMAC AR: pain subscale	WOMAC AR: stiffness subscale	WOMAC AR: difficulties subscale	Total WOMAC AR score subscale
Floor (%)	2.99%	2.99%	3.48%	1.49%	2.49%	23.88%	1.49%	1.00%
Ceiling (%)	1.99%	4.48%	0%	0%	1.49%	2.49%	1.99%	1.49%
Shapiro–Wilk	0.036	< 0.001	0.008	0.003	< 0.001	< 0.001	< 0.001	< 0.001
Minimum	0.00	0.00	0.00	0.00	0.00	0.00	0.00	0.00
Max	100.00	100.00	87.50	89.58	20.00	8.00	68.00	100.00
Median	50.00	62.50	43.75	54.17	8.00	2.00	29.00	40.63
IQ 1	31.25	37.50	31.25	35.42	4.00	1.00	14.00	20.83
IQ 3	62.50	81.25	56.25	64.58	12.00	4.00	40.00	56.25

Descriptive statistics for KOOS-12 AR and WOMAC-AR subscales and total scores in the full study sample. Values are presented as median and interquartile range (IQR). Floor and ceiling effects are expressed as percentages

*KOOS-12* Knee injury and Osteoarthritis Outcome Score–12 items, *KOOS-12 AR* Arabic version of the Knee injury and Osteoarthritis Outcome Score–12 items, *WOMAC* Western Ontario and McMaster Universities Osteoarthritis Index, *WOMAC-AR* Arabic version of the Western Ontario and McMaster Universities Osteoarthritis Index, *QOL* Quality of Life, *IQ* Interquartile

### Reliability/Consistency

- Internal consistency

The KOOS-12 AR summary scores (pain, function, QOL, and total) showed excellent coherence at the scale level. Across the three subscale scores and the total KOOS-12 AR score, Cronbach’s *α* was 0.93 and standardized Cronbach’s *α* was 0.93, indicating very high consistency between KOOS-12 AR domains. Pairwise correlations between the subscale scores and the total score were strong (*r* = 0.83–0.93), and correlations among subscales ranged from 0.60 to 0.77. Descriptively, in the full study sample (*N* = 201), mean KOOS-12 AR scores were 47.7 ± 22.4 for pain, 58.8 ± 26.7 for function, 43.3 ± 20.0 for QOL, and 49.9 ± 20.5 for the total score (Table 2). When the summary scores were considered jointly as components of a single scale, corrected component-total correlations were high (CITC range 0.73–1.00), and Cronbach’s *α* if a component was deleted ranged from 0.86 to 0.94, with no deletion producing a meaningful improvement in *α*, supporting retention of all four summary scores (Table 3).

b) Test–retest reliability

**Table 2:** KOOS-12 AR summary scores of the full study sample (*N* = 201)

Score	Possible range	Observed range	Mean ± SD
Pain subscale	0–100	0–100	47.7 ± 22.4
Function subscale	0–100	0–100	58.8 ± 26.7
QoL subscale	0–100	0–87.5	43.3 ± 20.0
Total KOOS-12 AR score	0–100	0–89.6	49.9 ± 20.5

Descriptive statistics for KOOS-12 AR subscales and total score in the full study sample. Possible score ranges, observed score ranges, and mean ± standard deviation (SD) are presented

*KOOS-12* Knee injury and Osteoarthritis Outcome Score–12 items, *KOOS-12 AR* Arabic version of the Knee injury and Osteoarthritis Outcome Score–12 items, *QOL* Quality of Life, *SD* Standard Deviation

**Table 3:** Internal consistency of KOOS-12 AR summary scores

	Cronbach’s *α*	Standardized *α*	CITC range	*α* if component deleted (range)
KOOS-12 AR summary scores (pain, function, QoL)	0.93	0.93	0.73–1.00	0.86–0.94

Internal consistency statistics for KOOS-12 AR summary scores, including Cronbach’s *α*, standardized Cronbach’s *α*, corrected item–total correlation (CITC) ranges, and Cronbach’s *α* if component deleted

*KOOS-12* Knee injury and Osteoarthritis Outcome Score–12 items, *KOOS-12 AR* Arabic version of the Knee injury and Osteoarthritis Outcome Score–12 items, *QOL* Quality of Life, *CITC* Corrected Item–Total Correlation

In the retest subgroup (*n* = 91), scores demonstrated excellent short-interval stability with no evidence of systematic change between assessments (Table 4). Mean differences between Time 1 and Time 2 were negligible across all subscales and for the total score, and none reached statistical significance.

**Table 4:** Short-interval stability of KOOS-12 AR scores in clinically stable participants (*n* = 91)

Score	Time 1 mean ± SD	Time 2 mean ± SD	Mean difference Δ (T2–T1)	95% CI for Δ	*p* (paired *t*-test)
Pain	46.2 ± 22.0	45.8 ± 22.5	−0.36	−1.00 to 0.28	0.262
Function	54.3 ± 25.8	54.3 ± 25.6	−0.02	−0.69 to 0.65	0.948
QoL	43.3 ± 20.0	42.9 ± 20.2	−0.33	−0.96 to 0.30	0.300
Total score	47.9 ± 20.4	47.7 ± 20.8	−0.24	−0.92 to 0.44	0.482

Test–retest stability results for KOOS-12 AR subscales and total score in clinically stable participants. Mean ± standard deviation (SD) at Time 1 and Time 2, mean change (Δ), 95% confidence intervals (CI) and *p*-values are presented

*KOOS-12* Knee injury and Osteoarthritis Outcome Score–12 items, *KOOS-12 AR* Arabic version of the Knee injury and Osteoarthritis Outcome Score–12 items, *QOL* Quality of Life, *SD* Standard Deviation, *CI* Confidence Interval, *Δ* Change score (Time 2 minus Time 1)

Test–retest reliability was excellent for all KOOS-12 AR scores, with single-measure ICC(2,1) values consistently above 0.98 (Table 5). Measurement error was low, with small standard errors of measurement (approximately 2–3 points on the 0–100 scale), corresponding to minimal detectable changes at the individual level of approximately 6 points.

**Table 5:** Test–retest reliability and measurement error of KOOS-12 AR scores (*n* = 91)

Score	ICC (2,1)	SEM (points)	MDC_individual (points)
Pain	0.990	2.15	5.96
Function	0.992	2.28	6.31
QoL	0.989	2.12	5.89
Total score	0.987	2.29	6.34

Test–retest reliability and measurement error indices for KOOS-12 AR subscales and total score. Intraclass correlation coefficients (ICC), standard error of measurement (SEM), and minimal detectable change at the individual level (MDC_individual) are presented

*KOOS-12* Knee injury and Osteoarthritis Outcome Score–12 items, *KOOS-12 AR* Arabic version of the Knee injury and Osteoarthritis Outcome Score–12 items, *ICC* Intraclass Correlation Coefficient, *ICC(2,1)* Two-way random-effects intraclass correlation coefficient for absolute agreement, single measures, *SEM* Standard Error of Measurement, *MDC* Minimal Detectable Change, *MDC_individual* Minimal Detectable Change at the individual level, *QOL* Quality of Life

**Fig. 2:**
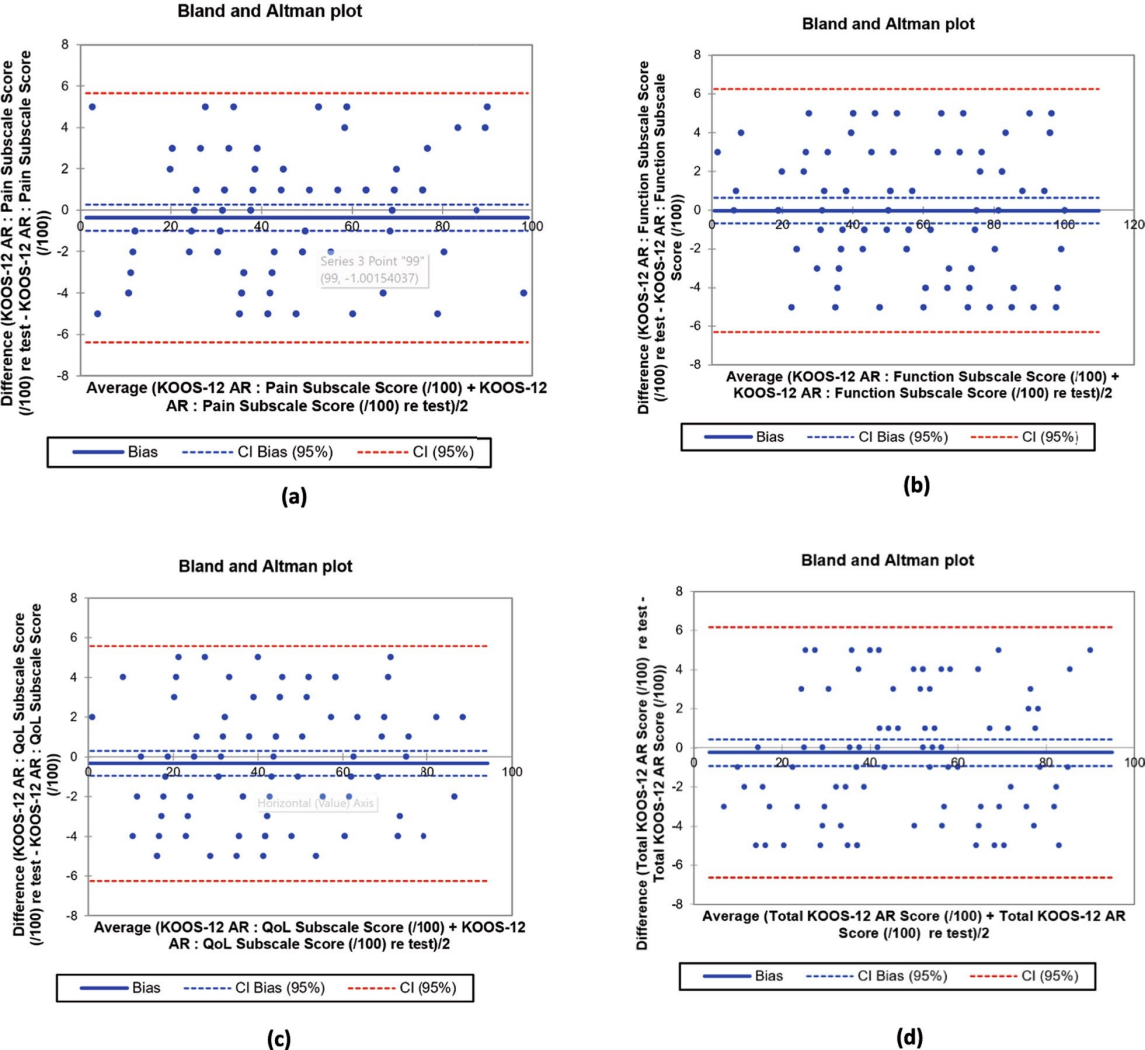
Bland–Altman plots for test–retest agreement of KOOS-12 AR scores (*n* = 91). **a** Pain subscale (/100), **b** Function subscale (/100), **c** QOL subscale (/100), and **d** Total KOOS-12 AR score (/100). Each plot displays the mean difference (bias, solid blue line), 95% confidence interval of the bias (dashed blue lines), and the 95% limits of agreement (dashed red lines)

Bland–Altman analyses showed negligible bias (Pain −0.36, Function −0.02, QOL −0.33, Total −0.24 points) and narrow 95% limits of agreement of about ± 6–7 points (Pain −6.4 to 5.7; Function −6.3 to 6.3; QOL −6.2 to 5.6; Total −6.6 to 6.2), with no relevant patterns suggesting proportional bias (Fig. 2). These results indicate high short-interval stability of KOOS-12 AR scores.

### Validity

- Content validity

Content validity was supported. All items were judged appropriate for the target construct and population, with only minor wording refinements made to improve clarity. Cognitive debriefing with 10 clinical patients identified no missing concepts and required no item removal.

b) Construct/structural validity

For each subscale, measures of sampling adequacy were acceptable and Bartlett’s tests of sphericity were highly significant, supporting the suitability of the data for factor analysis (Table 6). Each subscale demonstrated a clear one-component solution, with the first component explaining more than 60% of the variance in all cases, supporting the essential unidimensionality of the pain, function, and QOL subscales.

**Table 6:** Principal component analysis of KOOS-12 AR subscales at item level (*n* = 201)

Subscale	Number of items	KMO	Bartlett’s *χ*^2^ (df)	*p*-value	First eigenvalue	Variance explained by first component (%)	Range of loadings on first component
Pain	4	0.778	244.494 (6)	< 0.0001	2.500	62.5	0.455–0.541
Function	4	0.784	329.210 (6)	< 0.0001	2.716	67.9	0.472–0.530
QoL	4	0.749	281.541 (6)	< 0.0001	2.485	62.1	0.542–0.891

Principal component analysis (PCA) results for KOOS-12 AR subscales at the item level. Number of items, Kaiser–Meyer–Olkin (KMO) measure, Bartlett’s test of sphericity (*χ*^2^, df), first eigenvalue, percentage of variance explained, and range of standardized factor loadings are presented

*KOOS-12* Knee injury and Osteoarthritis Outcome Score–12 item, *KOOS-12 AR* Arabic version of the Knee injury and Osteoarthritis Outcome Score–12 items, *PCA* Principal Component Analysis, *KMO* Kaiser–Meyer–Olkin measure of sampling adequacy, *QOL* Quality of Life, *χ*^*2*^ Chi-square, *df* Degrees of freedom

When all 12 items were analyzed together, overall sampling adequacy was excellent and Bartlett’s test remained highly significant, confirming the suitability of the data for factor analysis (Table 7). Although eigenvalue criteria suggested more than one component, the first component was interpreted as dominant because it explained more than 50% of the total variance, exceeding commonly accepted thresholds for a general factor in PROM validation [19], while subsequent components accounted for substantially smaller proportions of variance. All items showed moderate loadings on this dominant first component, with additional clustering by their intended domains across the next components, indicating a strong general “knee impact” factor alongside distinguishable pain, function, and QOL dimensions. This pattern supports the structural validity of the KOOS-12 AR both at the subscale level and for an overall composite score.

c) Convergent validity

**Table 7:** Principal component eigenvalues for the 12-item KOOS-12 AR (*n* = 201)

Component	Eigenvalue	Variance explained (%)	Cumulative variance (%)
Pain—How often	6.076	50.6	50.6
Pain—Walking on a flat surface	1.182	9.9	60.5
Pain—Going up or down stairs	0.985	8.2	68.7
Pain—Sitting or lying	0.681	5.7	74.4
Function, daily living—Rising from sitting	0.575	4.8	79.2
Function, daily living—Standing	0.497	4.1	83.3
Function, daily living—Getting in/out of a car	0.443	3.7	87.0
Function, daily living—Twisting/pivoting	0.400	3.3	90.3
Quality of Life—Awareness	0.339	2.8	93.2
Quality of Life—Life style modification	0.299	2.5	95.6
Quality of Life—Lack of confidence	0.264	2.2	97.8
Quality of Life—Difficulty in general	0.260	2.2	100.0

Eigenvalues, percentage of variance explained, and cumulative variance for components derived from principal component analysis (PCA) of the 12 KOOS-12 AR items

*KOOS-12* Knee injury and Osteoarthritis Outcome Score–12 items, *KOOS-12 AR* Arabic version of the Knee injury and Osteoarthritis Outcome Score–12 items, *PCA* principal component analysis

**Fig. 3:**
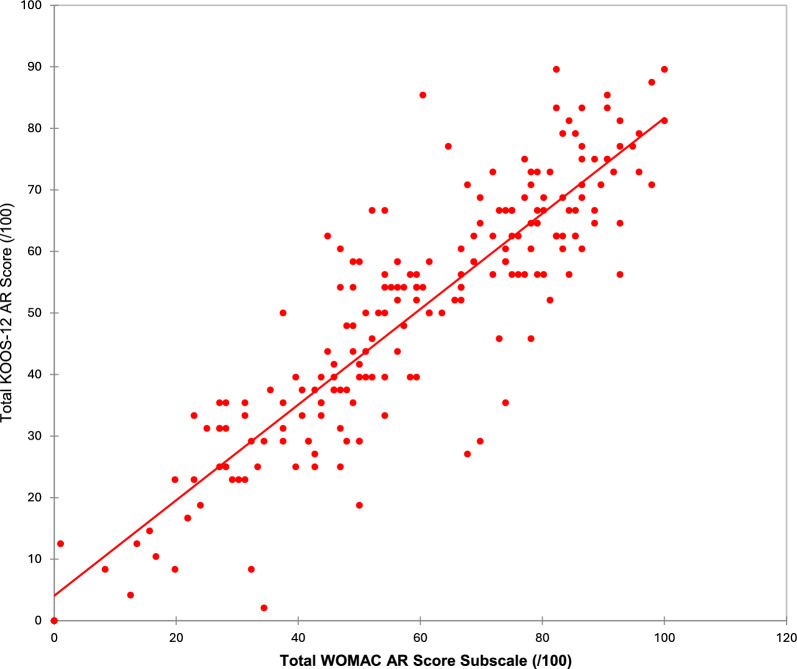
Convergent validity: KOOS-12 AR Total WOMAC-AR Total (Spearman *ρ* = 0.879; *p* < 0.0001)

Convergent validity of the KOOS-12 AR was excellent. As hypothesized, the total KOOS-12 AR score showed a strong positive correlation with the WOMAC-AR total score after transformation to a common 0–100 scale (Spearman’s *ρ* = 0.879, *p* < 0.0001; Fig. 3), indicating substantial overlap between the constructs measured by the two instruments.

## Discussion

The present study demonstrated excellent psychometric properties of the KOOS-12 AR. Internal consistency was high across the pain, function, QOL, and total scores, with Cronbach’s *α* = 0.93, indicating strong coherence among domains. Test–retest reliability was likewise excellent, with ICC(2,1) values ranging from 0.987 to 0.992 across subscales, reflecting highly stable scores over the 5–10 day interval. Measurement error indices were small, and Bland–Altman analyses showed minimal bias with narrow limits of agreement, confirming reproducibility. Convergent validity was supported by a strong correlation between the KOOS-12 AR Total score and the WOMAC-AR Total score (Spearman’s *ρ* = 0.879), demonstrating substantial alignment with an established, widely used measure of knee osteoarthritis. These findings underscore the suitability of KOOS-12 AR for widespread adoption in Arabic-speaking countries as a dependable and feasible tool for use in both clinical practice and research across diverse patient groups. Notably, these benefits are not restricted to a specific geographic region but extend to Arabic-speaking patients worldwide.

Additionally, the results of this study are widely consistent with the original KOOS-12 validation [8, 10, 20]. As reported by Gandek et al., this score shows strong internal consistency, with all subscales exceeding 0.70 and the summary score > 90. It demonstrated satisfactory construct validity and responsiveness across the pain, function, and QOL subscales, with results comparable to those of the full-length KOOS scales. In the original validation studies, the KOOS-12 summary score also demonstrated strong responsiveness and discriminative ability across clinically relevant patient groups, further supporting its robustness relative to the full-length KOOS [10]. This study therefore underscores strong consistency with the validity and reliability of the KOOS-12 in other populations. Notably, the magnitude of reliability estimates and the clear unidimensional structure observed in this Arabic-speaking cohort are comparable to those reported in Western and Asian populations, despite differences in language, cultural context, and clinical setting [21, 22]. The strong general factor observed across all 12 items may reflect the relatively homogeneous clinical presentation of knee osteoarthritis in this outpatient sample, which primarily included middle-aged and older adults. In contrast to prior validations conducted mainly in post–total knee replacement populations, these findings extend the applicability of the KOOS-12 to a nonsurgical osteoarthritis population and support its use across a broader range of disease severity. In this context, language-specific PROM validations reflect the global scope of contemporary orthopedic research, emphasizing linguistic equivalence as a prerequisite for methodological rigor and cross-cultural comparability [23]. Accordingly, the KOOS-12 AR broadens the reach of a widely used international instrument to one of the largest linguistic populations worldwide.

In contrast to other PROMs such as the knee and hip health-related QOL questionnaire (Mini-OAKHQOL), the Knee Outcome Survey–Sports Activities Scale (KOS-SAS), the Oxford Knee Score (OKS), and the full-length KOOS, the KOOS-12 has not yet been translated into Arabic [24–27]. Compared with the full-length KOOS, the KOOS-12 imposes minimal burden on respondents while offering excellent responsiveness [8], highlighting the advantage of translating and validating the KOOS-12 into Arabic. This score has already been translated into Chinese, Sinhala, and Persian [28–30], demonstrating its clinical applicability, psychometric strength, and high perceived value as a globally recognized tool for knee injury and osteoarthritis evaluation and follow-up. Given the large Arabic-speaking population worldwide [14], a validated Arabic KOOS-12 is needed to avoid both restricted inclusion and under-representation in global research. Lacking this efficient tool places Arabic-speaking patients at a clear disadvantage.

More broadly, orthopedic research conducted in Arabic-speaking regions has increasingly incorporated PROMs; however, in several musculoskeletal subspecialties, investigators have relied on nonvalidated translations or English-language instruments owing to the limited availability of formally validated Arabic PROMs [31, 32]*.* By contrast, multiple PROMs outside the knee domain, such as Arabic versions of shoulder, spine, and general health outcome measures [33–35], have been successfully validated and subsequently adopted in regional and international research, illustrating a clear demand for linguistically appropriate tools. This trend is further supported by our screening of PubMed-published studies conducted in primary Arabic-speaking countries over the past 3 years, showing that fewer than approximately 5% used a validated patient-reported outcome measure, suggesting sustained methodological interest in standardized scoring systems. These observations align with the findings of Al-Ebrahim et al. in 2023 [32], who systematically reviewed 260 studies encompassing 317 Arabic PROMs and reported that 83.8% evaluated psychometric properties and 75.8% involved cross-cultural adaptation, most commonly through forward and back-translation. Notably, however, only 1 of the 317 PROMs (less than 0.3%) fulfilled criteria for optimal cross-cultural adaptation and psychometric quality, highlighting that a substantial proportion of Arabic PROM use remains methodologically limited or based on sub-optimally validated instruments. Nevertheless, a dedicated bibliometric analysis with prespecified inclusion criteria and broader search strategies would be required to precisely quantify the proportion of Arabic-region orthopedic literature incorporating scoring systems and to distinguish validated instruments from non-validated adaptations.

Importantly, Arabic-speaking populations are not confined to the Middle East and North Africa but include substantial communities in North America, particularly in the USA, where Arabic is among the fastest-growing spoken languages [36]*.* In U.S. Census Bureau estimates, approximately 1.4 million individuals report speaking Arabic at home [37]. Therefore, the lack of validated Arabic PROMs risks systematic exclusion and measurement bias not only in regional studies but also in USA-based and multinational clinical research. A validated KOOS-12 AR directly addresses this gap by enabling linguistically appropriate assessment and equitable representation of Arabic-speaking patients.

Moreover, while objective clinical assessments such as range of motion, muscle strength, swelling, and gait analysis constitute essential metrics for evaluating knee injury and osteoarthritis, a patient’s viewpoint and personal experience often remain underdocumented. By complementing these objective outcomes, PROMs fill critical assessment gaps. Consistently, surgeons regard them as crucial in patient care, as they improve functional outcomes and postoperative recovery [38, 39]. In the orthopedic field in particular, PROMs have gained increasing recognition over the years [40, 41]. They are considered by the American Academy of Orthopedic Surgeons (AAOS) to be among the best measures of the success of an orthopedic procedure [42]. When PROM-based monitoring is implemented, improvements in health-related QOL and reductions in depressive symptoms have been observed among knee replacement patients [7]. As a result, the KOOS-12 AR will help achieve inclusion and comparability in knee-centered orthopedic care across Arabic-speaking regions. By identifying patients who lack subjective improvement, it can serve as a valuable guide in clinical settings and a reliable tool for decision-making, thereby contributing to enhanced quality of care within these populations.

Consequently, the KOOS-12 AR was developed in accordance with established international validation standards, supporting broad cross-regional applicability. With regard to validation initiatives in Arabic-speaking countries, the present study itself represents the first coordinated effort to formally translate, culturally adapt, and psychometrically validate the KOOS-12 in Arabic in accordance with internationally accepted PROM guidelines. This initiative was conducted in clinical settings within Arabic-speaking countries, involved multidisciplinary clinical and methodological expertise, and followed a rigorous process including forward–backward translation, expert consensus review, pilot testing, and comprehensive psychometric evaluation, thereby facilitating its use in future regional clinical studies, multicenter collaborations, and international registries.

However, this study presents some limitations. It was conducted exclusively in two clinical centers, which may limit generalizability across Arabic-speaking regions. Although adequate for initial validation, the sample size could restrict subgroup analyses. Finally, some cultural and dialectal nuances may remain untested. Nonetheless, the questionnaire was translated into Modern Standard Arabic (Foṣḥa), the official form of the language taught and understood throughout Arabic-speaking countries, thereby ensuring broad linguistic comprehensibility despite regional variations.

## Conclusions

The present study establishes the first formally validated Arabic version of the KOOS-12. The KOOS-12 AR demonstrated excellent reliability, validity, structural coherence, and measurement reproducibility, confirming its psychometric robustness. It represents a culturally appropriate, linguistically accurate, and feasible instrument for assessing knee pain, function, and quality of life in Arabic-speaking patients with knee osteoarthritis or injury. It additionally supports more accurate evaluation, improved follow-up and QOL, and equitable inclusion of Arabic-speaking patients in multinational clinical studies and international orthopedic research.

## Supplementary Information

Supplementary material 1: Figure 1. The Arabic version of the KOOS-12 (KOOS-12 AR).

Supplementary material 2.

## Data Availability

No datasets were generated or analyzed during the current study.

## Abbreviations

KOOS-12: Knee injury and Osteoarthritis Outcome Score–12 items
KOOS-12 AR: Arabic version of the Knee injury and Osteoarthritis Outcome Score–12 items
KOOS: Knee injury and Osteoarthritis Outcome Score
QOL: Quality of life
WOMAC: Western Ontario and McMaster Universities Osteoarthritis Index
WOMAC-AR: Arabic version of the Western Ontario and McMaster Universities Osteoarthritis Index
PROMs: Patient-reported outcome measures
ICC: Intraclass correlation coefficient
ICC(2,1): Two-way random-effects intraclass correlation coefficient for absolute agreement, single measures
SEM: Standard error of measurement
MDC: Minimal detectable change
MDC_individual: Minimal detectable change at the individual level
MDC_group: Minimal detectable change at the group level
PCA: Principal component analysis
KMO: Kaiser–Meyer–Olkin measure of sampling adequacy
CI: Confidence interval
IQR: Interquartile range
CITC: Corrected item–total correlation
SD: Standard deviation
Δ (Delta): Change score (Time 2 minus Time 1)
AAOS: American Academy of Orthopaedic Surgeons
OKS: Oxford knee score
KOS-SAS: Knee Outcome Survey–Sports Activities Scale
Mini-OAKHQOL: Mini osteoarthritis knee and hip quality of life questionnaire
MAPI: MAPI research trust
IRB: Institutional Review Board
