# Supplementary material for: Cross-cultural adaptation and psychometric validation of the first Arabic KOOS-12: a reliable tool for assessing knee outcomes in Arabic-speaking populations
Source: Knee Surg Relat Res. 2026 Apr 10;38:16. doi: 10.1186/s43019-026-00316-6 (PMC13067642; doi:10.1186/s43019-026-00316-6)
Supplement: Supplementary file 1 — Supplementary material 1: Figure 1. The Arabic version of the KOOS-12 (KOOS-12 AR). [file 43019_2026_316_MOESM1_ESM.docx]

**درﺟﺔ إﺻﺎﺑﺔ اﻟﺮﻛﺒﺔ وﻧﺘﺎﺋﺞ اﻟﺘﮭﺎب اﻟﻤﻔﺎﺻﻞ اﻟﻌﻈﻤﻲ-12**

**معلومات عامة -I**

_______________________اسم المريض :

_______________________تاريخ الولادة :

الجنس: ☐ ذكر ☐ أنثى

_______________________رقم الهاتف :

المهيمنة: ☐ يمين ☐ يسار ☐ كليهماالسّاق

| **اﻟﺘﻌﻠﯿﻤﺎت: ﯾﺴﺘﻔﺴﺮ ھﺬا اﻻﺳﺘﺒﯿﺎن ﻋﻦ آراﺋﻚ ﺣﻮل رﻛﺒﺘﻚ. ﯾﺮﺟﻰ اﻹﺟﺎﺑﺔ ﻋﻠﻰ ﻛﻞ ﺳﺆال ﻣﻦ ﺧﻼل اﺧﺘﯿﺎر اﻹﺟﺎﺑﺔ اﻟﻤﻨﺎﺳﺒﺔ، ﺑﺤﯿﺚ .ﺗﻜﻮن ھﻨﺎك إﺟﺎﺑﺔ واﺣﺪة ﻟﻜﻞ ﺳﺆال. إذا ﻛﻨﺖ ﻏﯿﺮ ﻣﺘﺄﻛﺪ ﻣﻦ ﻛﯿﻔﯿﺔ اﻹﺟﺎﺑﺔ ﻋﻠﻰ ﺳﺆال ﻣﺎ، ﯾﺮﺟﻰ إﻋﻄﺎء أﻓﻀﻞ إﺟﺎﺑﺔ ﯾﻤﻜﻨﻚ ﺗﻘﺪﯾﻤﮭﺎ** | |
| --- | --- |
| **اﻷﻟﻢ** | |
| **ﻛﻢ ﻣﺮة ﺗﺸﻌﺮ ﺑﺄﻟﻢ ﻓﻲ اﻟﺮﻛﺒﺔ؟ :**  ☐ 0 ☐ 1 ☐ 2 ☐ 3 ☐ 4 | **0 (أﺑﺪًا)، 1 (ﺷﮭﺮﯾًﺎ)، 2 (أﺳﺒﻮﻋﯿًﺎ)،**  **3 (ﯾﻮﻣﯿًﺎ)، 4 (داﺋﻤًﺎ)** |
| **ﻣﺎ ﻣﻘﺪار اﻷﻟﻢ ﻓﻲ اﻟﺮﻛﺒﺔ اﻟﺬي ﺷﻌﺮت ﺑﮫ ﺧﻼل اﻷﺳﺒﻮع اﻟﻤﺎﺿﻲ أﺛﻨﺎء اﻟﻘﯿﺎم ﺑﺎﻷﻧﺸﻄﺔ اﻟﺘﺎﻟﯿﺔ؟ :** | |
| **النشاط** | **0 (لا يوجد)، 1 (خفيف)، 2 (متوسط)، 3 (شديد)، 4 (شديد للغاية)** |
| اﻟﻤﺸﻲ ﻋﻠﻰ أرض ﻣﺴﺘﻮﯾﺔ | ☐ 0 ☐ 1 ☐ 2 ☐ 3 ☐ 4 |
| ﺻﻌﻮد أو ﻧﺰول اﻟﺴﻼﻟﻢ | ☐ 0 ☐ 1 ☐ 2 ☐ 3 ☐ 4 |
| اﻟﺠﻠﻮس أو اﻻﺳﺘﻠﻘﺎء | ☐ 0 ☐ 1 ☐ 2 ☐ 3 ☐ 4 |
| **اﻟﻮظﯿﻔﺔ، اﻷﻧﺸﻄﺔ اﻟﯿﻮﻣﯿﺔ** | |
| **اﻷﺳﺌﻠﺔ اﻟﺘﺎﻟﯿﺔ ﺗﺘﻌﻠﻖ ﺑﻮظﯿﻔﺘﻚ اﻟﺠﺴﺪﯾﺔ. ﺑﻤﻌﻨﻰ ذﻟﻚ ﻗﺪرﺗﻚ ﻋﻠﻰ اﻟﺘﺤﺮك واﻻﻋﺘﻨﺎء ﺑﻨﻔﺴﻚ. ﻟﻜﻞ ﻣﻦ اﻷﻧﺸﻄﺔ اﻟﺘﺎﻟﯿﺔ، ﯾﺮﺟﻰ ﺗﺤﺪﯾﺪ درﺟﺔ اﻟﺼﻌﻮﺑﺔ اﻟﺘﻲ واﺟﮭﺘﮭﺎ ﺧﻼل اﻷﺳﺒﻮع اﻟﻤﺎﺿﻲ ﺑﺴﺒﺐ رﻛﺒﺘﻚ:** | |
| **النشاط** | **0 (لا يوجد)، 1 (خفيف)، 2 (متوسط)، 3 (شديد)، 4 (شديد للغاية)** |
| اﻟﻨﮭﻮض ﻣﻦ وﺿﻊ اﻟﺠﻠﻮس | ☐ 0 ☐ 1 ☐ 2 ☐ 3 ☐ 4 |
| اﻟﻮﻗﻮف | ☐ 0 ☐ 1 ☐ 2 ☐ 3 ☐ 4 |
| اﻟﺪﺧﻮل إﻟﻰ اﻟﺴﯿﺎرة / اﻟﺨﺮوج ﻣﻦ اﻟﺴﯿﺎرة | ☐ 0 ☐ 1 ☐ 2 ☐ 3 ☐ 4 |
| اﻟﺘﺤﺮك ﺑﻄﺮﯾﻘﺔ ﺗﺘﻄﻠﺐ اﻟﺘﻮاء أو ﺗﺪوﯾﺮ اﻟﺮﻛﺒﺔ اﻟﻤﺼﺎﺑﺔ | ☐ 0 ☐ 1 ☐ 2 ☐ 3 ☐ 4 |
| **ﺟﻮدة اﻟﺤﯿﺎة** | |
| **ﻛﻢ ﻣﺮة ﺗﺸﻌﺮ ﺑﻮﺟﻮد ﻣﺸﻜﻠﺔ ﻓﻲ رﻛﺒﺘﻚ؟:**  ☐ 0 ☐ 1 ☐ 2 ☐ 3 ☐ 4 | **(أﺑﺪًا)، 1 (ﺷﮭﺮﯾًﺎ)، 2 (أﺳﺒﻮﻋﯿًﺎ)، 0**  **3 (ﯾﻮﻣﯿًﺎ)، 4 (باستمرار)** |
| **: ھﻞ ﻗﻤﺖ ﺑﺘﻌﺪﯾﻞ ﻧﻤﻂ ﺣﯿﺎﺗﻚ ﻟﺘﺠﻨﺐ اﻷﻧﺸﻄﺔ اﻟﺘﻲ ﻗﺪ ﺗﻀﺮ ﺑﺮﻛﺒﺘﻚ؟**  ☐ 0 ☐ 1 ☐ 2 ☐ 3 ☐ 4 | **0 (ﻋﻠﻰ اﻹطﻼق)، 1 (ﺑﺸﻜﻞ ﺧﻔﯿﻒ)،**  **2 (ﺑﺸﻜﻞ ﻣﻌﺘﺪل)، 3 (ﺑﺸﻜﻞ ﺷﺪﯾﺪ)،**  **4 (ﺑﺸﻜﻞ ﻛﺎﻣﻞ)** |
| **: إﻟﻰ أي ﻣﺪى ﺗﺆﺛﺮ ﻗﻠﺔ اﻟﺜﻘﺔ ﻓﻲ رﻛﺒﺘﻚ ﻋﻠﻰ ﺣﯿﺎﺗﻚ؟**  ☐ 0 ☐ 1 ☐ 2 ☐ 3 ☐ 4 | **0 (ﻋﻠﻰ اﻹطﻼق)، 1 (ﺑﺸﻜﻞ ﺧﻔﯿﻒ)،**  **2 (ﺑﺸﻜﻞ ﻣﻌﺘﺪل)، 3 (ﺑﺸﻜﻞ ﺷﺪﯾﺪ)،**  **4 (ﺑﺸﻜﻞ ﻛﺎﻣﻞ)** |
| **: ﺑﺸﻜﻞ ﻋﺎم، ﻛﻢ ﺗﻮاﺟﮫ ﻣﻦ ﺻﻌﻮﺑﺔ ﻣﻊ رﻛﺒﺘﻚ؟**  ☐ 0 ☐ 1 ☐ 2 ☐ 3 ☐ 4 | **0 (ﻋﻠﻰ اﻹطﻼق)، 1 (ﺑﺸﻜﻞ ﺧﻔﯿﻒ)،**  **2 (ﺑﺸﻜﻞ ﻣﻌﺘﺪل)، 3 (ﺑﺸﻜﻞ ﺷﺪﯾﺪ)،**  **4 (ﺑﺸﻜﻞ ﻛﺎﻣﻞ)** |
