# Supplementary material for: Cross-cultural adaptation and psychometric validation of the first Arabic KOOS-12: a reliable tool for assessing knee outcomes in Arabic-speaking populations
Source: Knee Surg Relat Res. 2026 Apr 10;38:16. doi: 10.1186/s43019-026-00316-6 (PMC13067642; doi:10.1186/s43019-026-00316-6)
Supplement: Supplementary file 2 — Supplementary material 2. [file 43019_2026_316_MOESM2_ESM.docx]

**Supplementary Table 1.** ***Cross-cultural adaptation of the KOOS-12 from English to Arabic***

| **Domain** | **Item No.** | **Original item (English, paraphrased)** | **Arabic version (back-translated into English)** | **Cultural / linguistic adaptation** | **Rationale** |
| --- | --- | --- | --- | --- | --- |
| **Pain** | 1 | Frequency of knee pain | How often do you experience pain in your knee? | None | Direct conceptual equivalence |
|  | 2 | Knee pain during walking on a flat surface | Amount of knee pain when walking on a flat surface | None | Universally understood daily activity |
|  | 3 | Knee pain when going up or down stairs | Amount of knee pain when ascending or descending stairs | None | Culturally neutral activity |
|  | 4 | Knee pain during sitting or lying | Amount of knee pain while sitting or lying down | Minor wording refinement | Clarified posture-related phrasing |
| **Function (daily living)** | 5 | Difficulty rising from a seated position | Degree of difficulty standing up from sitting | None | Equivalent functional activity |
|  | 6 | Difficulty standing | Degree of difficulty standing | None | Direct translation possible |
|  | 7 | Difficulty getting in or out of a car | Degree of difficulty entering or exiting a car | None | Activity applicable across cultures |
|  | 8 | Difficulty twisting or pivoting on the knee | Degree of difficulty twisting or pivoting on the knee | Minor wording refinement | Improved clarity in Modern Standard Arabic |
| **Quality of Life** | 9 | Awareness of knee problem | How often are you aware of your knee problem? | None | Conceptually identical |
|  | 10 | Lifestyle modification to protect the knee | Have you changed your lifestyle to avoid activities that may harm your knee? | Minor semantic refinement | Improved clarity without construct change |
|  | 11 | Lack of confidence in the knee | How much does lack of confidence in your knee trouble you? | None | Psychological construct preserved |
|  | 12 | Overall difficulty due to knee problem | Overall, how much difficulty does your knee cause you? | None | Direct conceptual equivalence |

**Legend:**

Item-by-item cross-cultural adaptation summary for the KOOS-12. For each item (by domain and item number), the table presents the English source item (paraphrased), the Arabic translation (shown via blinded back-translation into English), whether any cultural/linguistic adaptation was required, and the rationale for any modifications (semantic, idiomatic, experiential, or conceptual equivalence). “None” indicates no modification was needed.

**Abbreviations:**

**KOOS-12:** Knee injury and Osteoarthritis Outcome Score–12 items.

**KOOS-12 AR:** Arabic version of the Knee injury and Osteoarthritis Outcome Score–12 items.
